# Targeting the SARS-CoV-2 3CL^pro^ and NO/cGMP/PDE5 pathway in COVID-19: a commentary on PDE5 inhibitors

**DOI:** 10.2217/fca-2020-0201

**Published:** 2021-02-12

**Authors:** Milad Shirvaliloo

**Affiliations:** ^1^Faculty of Medicine, Tabriz University of Medical Sciences, Tabriz, Iran; ^2^Student Research Committee, Tabriz University of Medical Sciences, Tabriz, Iran

**Keywords:** 3CL^pro^, COVID-19, NO/cGMP, PDE5, PDE5 inhibitors, SARS-CoV-2, sildenafil, tadalafil, thromboembolism

Emerging in December 2019 in a local seafood market for the first time, coronavirus disease 2019 (COVID-19) is not merely an infection of the respiratory tract, but rather a systemic disease of cardiovascular ground, among others [1], which is of particular interest to this commentary. To date, among the many endeavors aimed at identification of novel treatments or repurposing of long-known medications for the treatment of COVID-19, several investigations have sought to delve into the efficacy of PDE5 inhibitors including sildenafil, tadalafil and vardenafil. Used predominantly in the treatment of male erectile dysfunction and pulmonary hypertension, PDE5 inhibitors may arguably inhibit the replication of severe acute respiratory syndrome coronavirus 2 (SARS-CoV-2), and prevent thromboembolism caused by inflammatory processes in COVID-19 patients.

The causative viral strain was soon found to share a 79 percent sequence identity with the original SARS-CoV, that was the strain responsible for the severe acute respiratory syndrome (SARS) outbreak in 2002 [2]. With the greater proportion of the genetic sequence identity occurring in the open reading frame regions, it could be easily anticipated that the envelope S-protein and ACE2 were going to be major players in SARS-CoV-2 pathogenesis [2,3]. This hypothesis was confirmed by Zhou *et. al*., when they reported successful *in vitro* infection of ACE2-expressing human cells by SARS-CoV-2 [4]. However, this was not the only mechanism involved in the pathogenesis of the novel coronavirus, as further investigations suggested the potential role of SARS-CoV-2 3CL^pro^ in the instigation of COVID-19 [5].

## The big picture: lesser-known mechanisms involved in SARS-CoV-2 pathogenesis

To the curious mind, however, the S-protein/ACE2 and viral protease mechanisms cannot be the sole culprits behind the deleterious inflammatory processes in COVID-19 patients. According to several studies published through the last decade, there is evidence indicating that certain pathways involving PDE5, particularly the NO/cGMP/PDE5 pathway, may aggravate these inflammatory processes in individuals with underlying metabolic diseases, for example, Type 2 diabetes mellitus [6,7]. This could also be the case with COVID-19, which is a systemic disease with an inflammatory background. Indeed, the mere inflammatory nature of COVID-19 cannot be considered as solid evidence for such speculation, but given the high expression of PDE5 in the lungs, one may begin to appraise the potential role of PDE5 in determining the prognosis of pulmonary inflammation caused by SARS-CoV-2 infection.

The idea came to fruition once US FDA approved the clinical application of nasal nitric oxide (NO), a pivotal molecule involved in the NO/cGMP/PDE5 pathway, for the treatment of interstitial pulmonary fibrosis associated with COVID-19 [8]. In spite of the beneficial effects of nasally inhaled NO in reversing pulmonary fibrosis, the systemic sequelae due to overproduction of NO, caused by the uncontrolled activation of iNOS in COVID-19, may not be in the best interest of patients. As one study suggests, excessive amounts of NO in the vasculature can lead to thromboembolism, a relatively common phenomenon observed in patients with severe COVID-19 [9]. This brings us to the therapeutic value of PDE5 inhibitors, particularly sildenafil and tadalafil, in the management of vascular sequelae caused by the inappropriate induction of NO/cGMP/PDE5 pathway.

Used commonly for the treatment of male erectile dysfunction, sildenafil is a PDE5 inhibitor that was originally introduced as an alternative to nitrates for relieving angina pectoris or chest pain [10,11]. The rationale behind this lied within the positive regulatory effects of sildenafil on the NO/cGMP pathway, later observed with tadalafil, as it inhibits the degradative action of PDE5 on the cGMP molecules expressed in the smooth muscle cells lining the vasculature. Accumulation of cGMP in these cells leads to dilation of vessels, and a concomitant increase in the blood flow to the sponge-like erectile tissue found in the penis. Due to the high expression of PDE5 in the arterioles of the lung, the primary organ involved in COVID-19, sildenafil and tadalafil have also been approved by the FDA for the treatment of pulmonary hypertension [12].

Through the last few years, the salutary effects of PDE5 inhibitors in the treatment of pulmonary arterial hypertension have further been supported by several studies. For instance, in 2017, one investigation conducted on 32 neonates with persistent pulmonary hypertension reported a significant decline in tricuspid regurgitation severity, and right ventricular end-diastolic diameter in two case groups of newborns treated discretely with sildenafil and tadalafil. The noteworthy aspect of this investigation was the comparable results achieved with sildenafil and tadalafil, indicating their near-equal efficacy [13]. These findings supported the results of a similar study, published 4 years earlier, that reported successful transition to a 40 mg daily dose of tadalafil in 97% of a population of 98 patients, the majority of whom had been receiving three 80–100 mg doses of sildenafil per day for at least a year [14].

## SARS-CoV-2 3CL^pro^: potential target for PDE5 inhibitors

Soon after the fulminant onset of the COVID-19 outbreak, early studies focused on the pathogenesis of SARS-CoV-2 reported the key role of S-protein and its interaction with the transmembrane ACE2 in the infection of host cell with the virus [2–4]. However, it was already well-established that coronaviruses produce polyproteins, that are further processed by certain viral proteases, particularly 3CL^pro^, into their functioning form, mediating the replication of the virus. Relevant evidence had been provided to support this speculation in 2004, long before the emergence of SARS-CoV-2; by a study that indicated the significantly lower mortality of patients with SARS, who had been treated with protease inhibitors (2.4 compared with 28.8%) [5]. Consistent with the findings regarding SARS-CoV, a recent study led by Jin *et. al.* reported the crystal structure of the SARS-CoV-2 3CL^pro^ [15].

This discovery prompted a group of scientists to conduct a computational study aimed at identifying potential inhibitors of 3CL^pro^. They calculated docking scores for several therapeutic agents to determine their binding affinity to the docking sites of 3CL^pro^. A more negative docking score indicated a higher affinity for binding with the main protease of SARS-CoV-2. Upon sorting the drugs based on their docking score, it was revealed that tadalafil and sildenafil, with a score of -9.3 and -8.9 kcal/mol, respectively, were actually potent inhibitors of 3CL^pro^. Similar results were also reported for vardenafil, and several other therapeutic agents, that are out of the scope of this paper. The study concluded that tadalafil, sildenafil and vardenafil might be promising therapeutic candidates for blocking the replication of SARS-CoV-2, with docking scores lower than -8.5 kcal/mol, that corresponded to <1 μM IC_50_ [16].

## NO/cGMP/PDE5: recognized pathway associated with inflammation in COVID-19

Not infrequently, patients with severe COVID-19 may develop certain life-threatening conditions, that necessitates inpatient management, and even admission to intensive care units. Thromboembolism is a known complication of COVID-19, which is partly attributed to the inordinate platelet aggregation in the vessels as a result of NO/cGMP/PDE5 pathway activation [17].

As a recent study led by Mario *et. al.* reports, infection with SARS-CoV-2 may lead to generation of exceedingly high amounts of NO, hence, the ultimate induction of NO/cGMP pathway [9]. Based on this theory, the acute lung failure observed in some patients is not solely driven by development of acute respiratory distress syndrome, but rather, microvascular thrombotic events in the vasculature of the lungs also appear to be a part of this vicious cycle. It is thought that COVID-19 could be associated with a disequilibrium in the production of NO by the iNOS and eNOS pathways. While the latter pathway contributes to NO-associated tissue protection, the former constitutes a known pro-inflammatory cascade that is usually triggered as a result of oxidative stress [9].

The clinically desirable activity of eNOS is partly mediated by AMPK; a serine-threonine protein kinase comprising three subunits [18]. It has been shown that AMPK counteracts inflammation through its inhibitory effects on iNOS. Supposedly, AMPK participates in formation of a long anti-inflammatory axis, that can be summarized as AMPK/eNOS/NO/cGMP. Interestingly, in one study published in 2015, administration of sildenafil to a group of patients with cerebellar demyelination resulted in downregulation of inactive AMPK and iNOS [19]; which may explain the potential efficacy of PDE5 inhibitors in blocking the iNOS-induced inflammation associated with COVID-19, and enhancing the AMPK/eNOS/NO/cGMP pathway to counteract thromboembolism in patients with underlying health conditions [9].

## Beyond hypotheses: investigations & clinical trials on PDE5 inhibitors

Published in June 2020, the DEDALO project is probably the first wide-scope study regarding the therapeutic efficacy of PDE5 inhibitors in diabetic patients with COVID-19. Short for “silDEnafil administration in DiAbetic and dysmetaboLic patients with COVID‐19”, this systematic review thoroughly explores all evidence indicating the involvement of NO/cGMP/PDE5 pathway in the pathogenesis of SARS-CoV-2. This pioneering study concluded that sildenafil may: counteract the downregulation of AT-1; downregulate the production of pro-inflammatory cytokines by monocytes, that are responsible for vascular damage in the pulmonary tissue; and inhibit the pathological transition of endothelial cells to mesenchymal cells, preventing thromboembolism in COVID-19 patients [20].

Initiated by Andrés Bello National University, “Sildenafil in COVID-19” is an ongoing clinical trial on a well-known PDE5 inhibitor. Expected to be completed by March 2021, this investigation seeks to evaluate two important prognostic parameters, arterial oxygenation and alveolar-arterial gradient, in COVID-19 patients with perfusion mismatch [21].

Last updated in March 2020, “A Pilot Study of Sildenafil in COVID-19” is another active clinical investigation on sildenafil, that has been designed to evaluate the therapeutic outcomes of a two-week treatment with 100 mg daily dose of sildenafil. Funded by Tongji Hospital, Qin Ning, China, this clinical trial aims to measure the rate of disease remission, along with the time and rate of entering the critical stage in an estimated population of ten patients with COVID-19 [22].

## Conclusion

Given the recent findings regarding the pathogenesis of SARS-CoV-2, and the pathways involved in COVID-19, resulting in pulmonary fibrosis and thromboembolism; it appears that PDE5 inhibitors could be good candidates in the clinical management of COVID-19 patients, especially those with a history of underlying cardiac and metabolic diseases. With their dual inhibitory effects on SARS-CoV-2 3CL^pro^ and the NO/cGMP/PDE5 pathway, PDE5 inhibitors suggestively act in more than one way against COVID-19, leading to the inhibition of viral replication and downregulation of pro-inflammatory pathways concerned with the induction of iNOS and instigation of thromboembolism. Nonetheless, it should be noted that the current insights are of preliminary nature, and further investigations need to be allocated to this pressing topic.
